# Prognosis Prediction and Surgical Benefit Subgroup Analysis in Anal Squamous Cell Carcinoma Patients Undergoing Concurrent Chemoradiotherapy

**DOI:** 10.1002/cam4.71091

**Published:** 2025-08-11

**Authors:** Quan Wang, Guangmin Wan, Lu Yang, Gang Xu

**Affiliations:** ^1^ Department of Radiation Oncology The Affiliated Cancer Hospital of Zhengzhou University & Henan Cancer Hospital Zhengzhou China

**Keywords:** anal squamous cell carcinoma, chemoradiotherapy, nomogram, OS, subgroup analysis

## Abstract

**Background:**

This study aimed to develop a nomogram to predict overall survival in anal squamous cell carcinoma (ASCC) patients undergoing chemoradiotherapy and to identify patients who may benefit from surgery.

**Methods:**

Data from 4697 ASCC patients were extracted from the Surveillance, Epidemiology, and End Results database. Of these, 2657 patients were included in the training set, 1136 patients in the internal validation set, and 904 patients in the external validation set. LASSO and Cox multivariate regression analyses were conducted to identify independent prognostic factors and construct the nomogram. The discriminatory performance of the nomogram was evaluated using the time‐dependent area under the receiver operating characteristic curve (ROC) and calibration curve. Decision curve analysis was used to compare the nomogram with the American Joint Committee on Cancer (AJCC) staging system. Additionally, a risk assessment system based on nomogram scores was developed. Propensity score matching and subgroup analysis were performed to identify groups with potentially better prognosis after surgery.

**Results:**

Four independent variables—age, sex, tumor size, and AJCC stage—were identified as key predictors for the nomogram. The nomogram demonstrated robust discriminatory ability, as evidenced by the time‐dependent ROC. Calibration plots showed strong concordance between the nomogram and real‐world data in the training and validation cohorts, with the nomogram outperforming the AJCC staging system. Patients were stratified into subgroups based on their risk scores, revealing significant differences in overall survival between the subgroups (*p* < 0.05). Subgroup analysis suggested that males might benefit from surgery, which was not observed in females.

**Conclusion:**

This nomogram could serve as a valuable tool for clinicians in predicting long‐term prognosis. We also identified patients who may benefit from surgery, providing a reference for treatment decisions in these patients.

## 1 Introduction

Anal squamous cell carcinoma (ASCC) is a relatively rare malignancy of the lower gastrointestinal tract, accounting for 85% of anal canal cancers (ACCs) in the United States [1, 2]. However, its incidence has risen in recent decades, partly due to the increasing number of human papillomavirus (HPV) infections. Over 90% of anal cancers are associated with HPV, highlighting the importance of addressing this disease [3, 4]. In terms of treatment, a recent study based on the National Cancer Database showed that the proportion of patients receiving local excision alone has increased over time in the United States, particularly for tumors smaller than 1 cm. This suggests that tiny tumors may be managed adequately with local excision alone; however, most patients are diagnosed with much larger tumors [5]. Currently, the National Comprehensive Cancer Network (NCCN) and European Society for Medical Oncology guidelines recommend CRT as the primary treatment for localized and locally advanced ASCC, with a 5‐year OS rate of 60% [6, 7, 8]. Furthermore, CRT as an initial treatment for stage IV patients with extra‐regional lymph node involvement but no distant metastasis has been associated with favorable OS outcomes compared to surgery [9]. Abdelazim et al. [10] also reported that CRT was associated with improved OS compared to chemotherapy alone. However, surgery is currently only salvage therapy in the management of ASCC [6, 7]. The efficacy of local excision versus chemoradiotherapy in stage I ASCC has been the focus of numerous studies, but the results remain inconsistent [11, 12].

Current studies have indicated that the AJCC staging system is no longer sufficient to predict overall survival in ASCC patients with complete accuracy [13, 14, 15]. Casadei‐Gardini et al. [15] retrospectively investigated the impact of laboratory indices on the treatment outcomes of anal cancer patients undergoing chemoradiotherapy. However, there is currently no universally accepted scoring system based on clinicopathologic characteristics, such as age, marital status, and other factors, to predict long‐term OS in this patient population. Therefore, the development of predictive models to assess treatment outcomes in ASCC patients undergoing CRT is essential for improving patient management. Nomograms, which integrate both clinical and pathological factors, provide a quantitative tool for predicting individual prognosis and treatment response [16]. These models have gained popularity in cancer research due to their ability to offer personalized predictions based on a patient's unique clinical and biological profile.

In this study, we aim to develop a nomogram to predict the prognosis of ASCC patients undergoing CRT. We strive to provide an evidence‐based framework to guide therapeutic decision‐making and enhance patient outcomes by integrating clinical, pathological, and treatment‐related variables. Additionally, we conducted a subgroup analysis to assess further the impact of surgery on the prognosis of ASCC.

## 2 Methods

### 2.1 Data Source and Patient Selection

**FIGURE 1:**
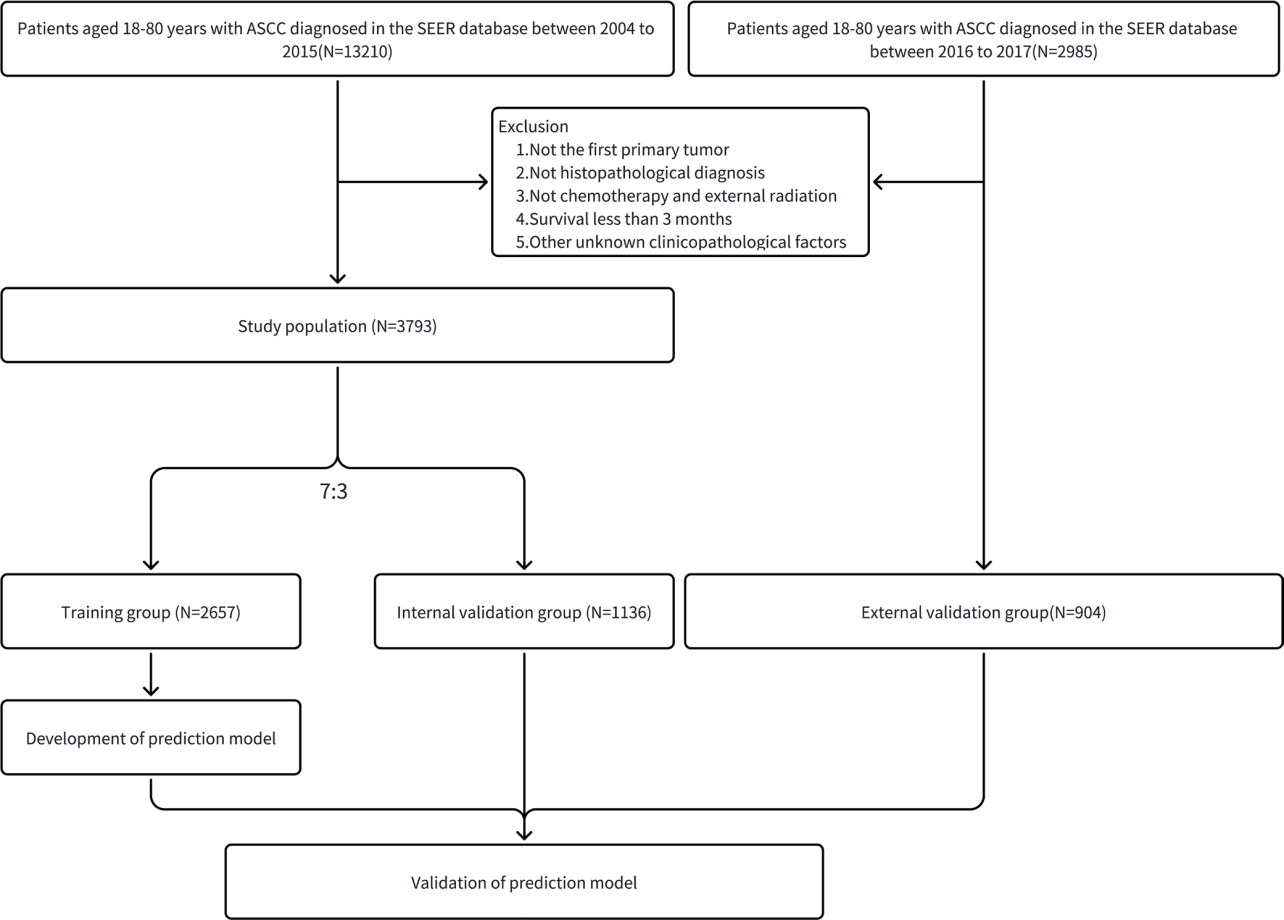
Flowchart of the patient screening.

The SEER data include the patients' registration number, personal information, site of the primary lesion, tumor information, and cause of death. Patients' data were downloaded from SEER*Stat version 8.4.4 (Research Data, 17 Registries, Nov 2023 Sub (2000–2021)). We identified cases of ASCC using the International Classification of Diseases for Oncology (ICD‐O) codes: 8051/3, 8052/3, 8070/3, 8071/3, 8072/3, 8073/3, 8074/3, 8075/3, 8076/3, 8078/3, 8083/3, 8094/3. Patients were categorized as having received CRT if they received chemotherapy and external beam radiation [11]. We determined eligible patients based on the following inclusion criteria: (a) those with lesion site recorded as anus, anal canal, and anorectum, (b) all patients were diagnosed with ASCC during 2004–2017, (c) patients were 18–80 years old. The exclusion criteria are as follows: (a) not histopathological diagnosis, (b) not chemotherapy and external beam radiation, (c) survival less than 3 months, (d) non‐first primary tumor, (e) incomplete clinicopathological information, including race, tumor grade, tumor size, AJCC stage, *T* stage, *N* stage, and *M* stage et al. The detailed screening procedure is shown in Figure 1.

### 2.2 Covariates

The following variables were included in the analysis: age at diagnosis, sex, marital status at diagnosis, race, primary tumor site, grade, tumor size, *T* stage, *N* stage, *M* stage, AJCC stage, surgery of primary tumors, chemotherapy, radiation, vital status, and survival months. The X‐tile software [17] was used to determine the appropriate cutoff values for the levels of age at diagnosis and tumor size. Age (years) was divided into < 61, 61–69, and > 69. Sex included male and female. Race was classified as white, black, or others, and marital status was dichotomized into married and unmarried (including separated, divorced, widowed). Tumor size (mm) was divided into < 25, 25–50, and > 50. Clinicopathological features, including histological grade, tumor size, tumor local situation (*T* stage), nodal involvement (*N* stage) and metastasis (*M* stage) were based on the 7th edition of the AJCC manual. Histological grade was classified into I, II, and III–IV, tumor local situation into *T*1, *T*2, *T*3, and *T*4, nodal involvement into *N*0 and *N*1, while *M* stage was divided into *M*0 and *M*1. Surgery information covered primary site surgery or not.

### 2.3 Statistical Analysis

A total of 4697 ASCC patients who met the inclusion and exclusion criteria were included in this study. Of these, 3973 patients from 2004 to 2015 were randomized in a 7:3 ratio into a training set and an internal validation set, while 904 patients from 2016 to 2017 were considered as an external validation set. These three datasets contained categorical data, presented as numbers and percentages, with intergroup comparisons conducted using the chi‐square test or Fisher's exact test. For the predictive model, LASSO regression was used to screen relevant variables. Multivariate Cox proportional hazards regression analysis was performed to identify independent prognostic factors, which were used to construct the nomogram. The nomogram was designed to predict survival rates at 5, 8, and 10 years, with risk scores for each independent prognostic factor calculated. We assessed the reliability and accuracy of the predictive model through time‐dependent receiver operating characteristic curves (ROC), the area under the curve (AUC), and calibration curves. Decision curve analysis (DCA) was employed to evaluate the practical value of the predictive model by estimating the net benefit under different risk thresholds. Risk stratification was performed based on the established nomogram, dividing patients into the low‐risk and high‐risk groups based on the cutoff value (87.08) of the nomogram score. Survival analysis was conducted using Kaplan–Meier curves, with log‐rank tests applied to compare survival curves. Additionally, PSM was performed using the “MatchIt” software package, with 1:1 nearest‐neighbor matching and a caliper of 0.001 for balanced comparisons based on surgical management. Statistical analysis was carried out using R software (version 4.4.2) (https://www.r‐project.org/), with statistical significance defined as a two‐sided *p* value of less than 0.05. OS, defined as the time from diagnosis to the most recent follow‐up or date of death, was the primary endpoint of this study. Ethical approval and informed consent were waived by the ethics committee due to the public availability of all data in the SEER database.

## 3 Results

### 3.1 Baseline Characteristics

Table 1 summarizes the demographic and clinicopathological characteristics of the training, internal validation, and external validation cohorts. The majority of patients are white, female, and no older than 69 years. The grade distribution primarily falls within the well and moderately differentiated categories. Additionally, approximately 10% of patients experienced adjacent organ involvement. Regarding the AJCC stage, most patients are in the relatively early stages. Approximately 65% of patients did not undergo surgery. Clinical characteristics of the training and internal validation sets are provided in Table S1. No significant differences were observed between the two groups (*p* > 0.05).

**TABLE 1:** The clinical characteristics of ASCC in the training set, internal validation set, and external validation set.

Variables	Training (*n* = 2657)	Internal validation (*n* = 1136)	External validation (*n* = 904)	*p*
Age (years)				
< 61	1583 (59.58)	681 (59.95)	450 (49.78)	< 0.0001
61–69	680 (25.59)	280 (24.65)	289 (31.97)	
> 69	394 (14.83)	175 (15.40)	165 (18.25)	
Sex				
Male	865 (32.56)	361 (31.78)	264 (29.20)	0.1737
Female	1792 (67.44)	775 (68.22)	640 (70.80)	
Race				
White	2357 (88.71)	1011 (89.00)	802 (88.72)	0.816
Black	233 (8.77)	99 (8.71)	85 (9.40)	
Others	67 (2.52)	26 (2.29)	17 (1.88)	
Size (mm)				
< 26	825 (31.05)	330 (29.05)	259 (28.65)	< 0.0001
26–50	1211 (45.58)	543 (47.80)	384 (42.48)	
> 50	621 (23.37)	263 (23.15)	261 (28.87)	
Grade				
I	274 (10.31)	127 (11.18)	96 (10.62)	< 0.0001
II	1295 (48.74)	542 (47.71)	457 (50.55)	
III–IV	1088 (40.95)	467 (41.11)	351 (38.83)	
Involved_organs				
No	2425 (91.27)	1027 (90.40)	803 (88.83)	0.0919
Yes	232 (8.73)	109 (9.60)	101 (11.17)	
AJCC				
I	494 (18.59)	203 (17.87)	141 (15.60)	0.0007
II	1127 (42.42)	477 (41.99)	332 (36.73)	
III	922 (34.70)	398 (35.04)	375 (41.48)	
IV	114 (4.29)	58 (5.11)	56 (6.19)	
*T*				
*T*1	597 (22.47)	237 (20.86)	172 (19.03)	0.0221
*T*2	1342 (50.51)	591 (52.02)	436 (48.23)	
*T*3	486 (18.29)	199 (17.52)	195 (21.57)	
*T*4	232 (8.73)	109 (9.60)	101 (11.17)	
*N*				
*N*0	1739 (65.45)	742 (65.32)	505 (55.86)	< 0.0001
*N*1	326 (12.27)	125 (11.00)	128 (14.16)	
*M*				
*M*0	2543 (95.71)	1078 (94.89)	849 (93.92)	0.0838
*M*1	114 (4.29)	58 (5.11)	55 (6.08)	
Surgery				
Yes	939 (35.34)	386 (33.98)	279 (30.86)	0.049
No	1718 (64.66)	750 (66.02)	625 (69.14)	

### 3.2 Independent Prognosis Factors

**FIGURE 2:**
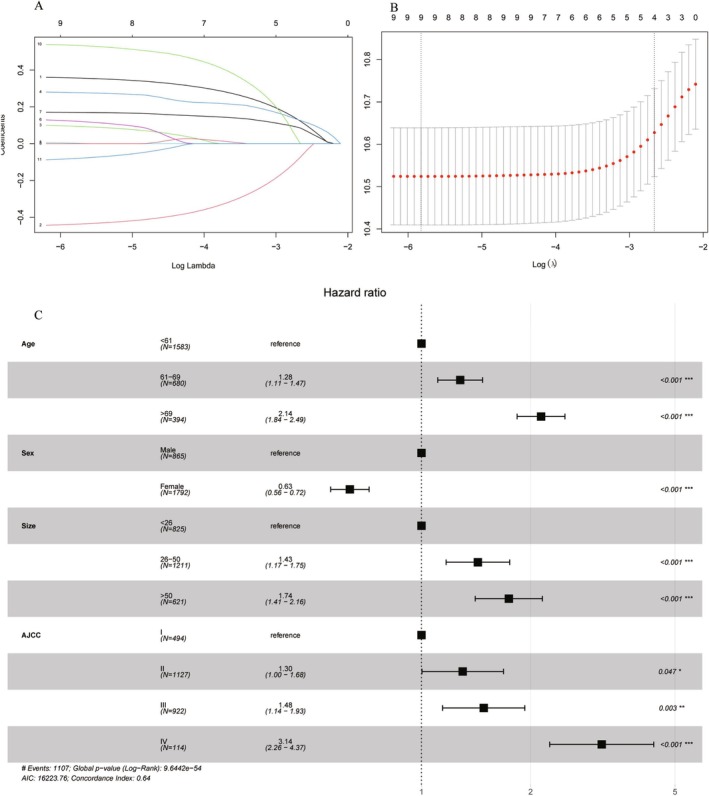
The distribution of LASSO coefficients for all variables of ASCC and four variables that were identified by LASSO regression analysis (A, B); forest plot demonstrating the multivariate Cox regression model for predicting OS in the development cohort (C).

LASSO analysis (Figure 2A,B) was performed to identify potential risk factors while preventing overfitting and penalizing the absolute values of regression coefficients in the training set. The large penalties caused estimates of weaker factors to shrink toward zero, leaving only the most influential factors in the model. The training set was analyzed using LASSO regression and multivariate analysis to identify the most significant predictors, which included age, sex, tumor size, and AJCC stage. The forest plot (Figure 2C) illustrates the multivariate Cox regression model for predicting OS in the training group. The results indicated that older age, male sex, larger tumor size, and higher AJCC stage were independent risk factors for patients.

### 3.3 Development of Nomogram

**FIGURE 3:**
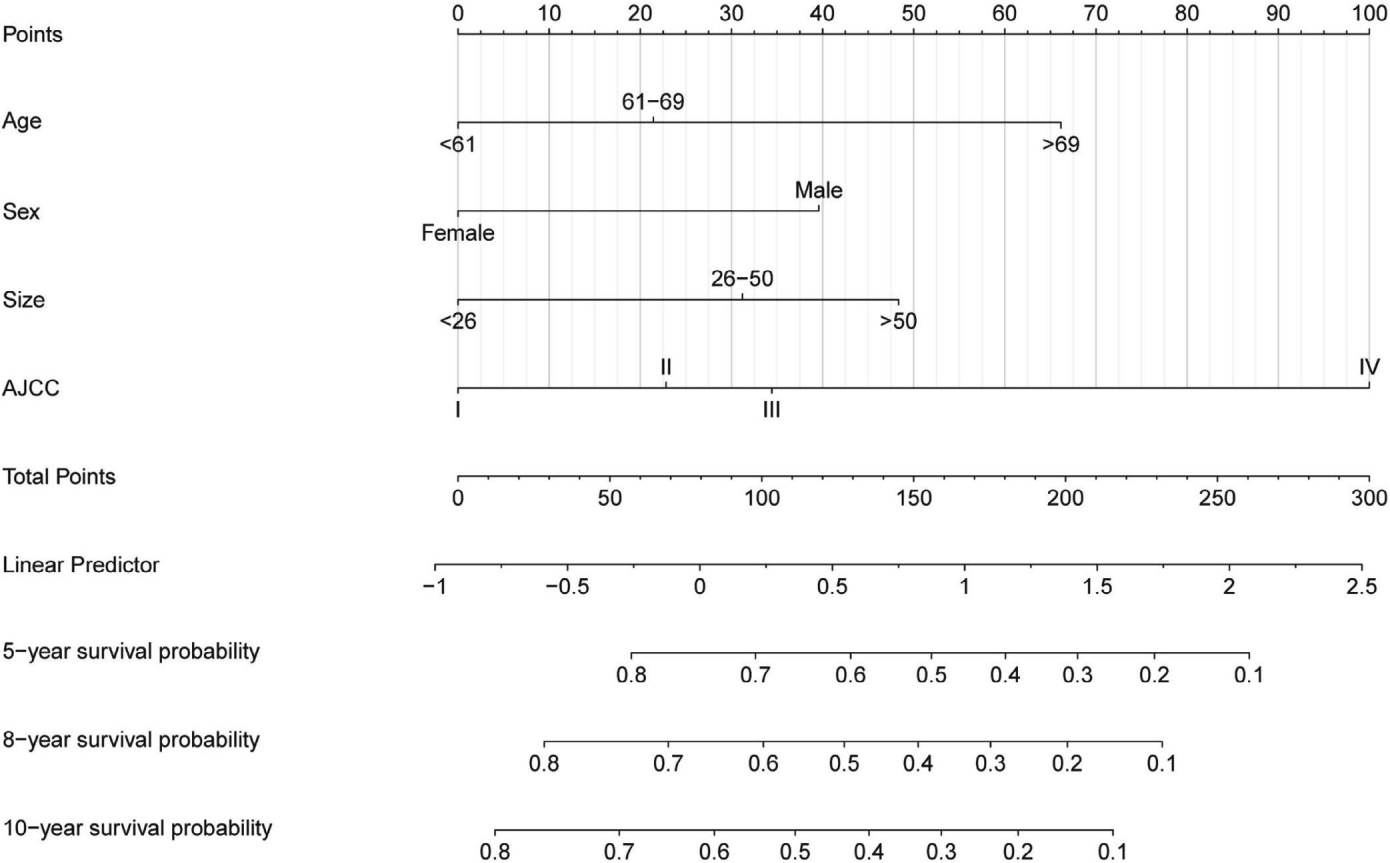
Nomogram for predicting 5‐, 8‐, and 10‐year OS in ASCC patients treated with chemoradiotherapy.

We constructed a traditional nomogram (Figure 3) based on the results of multivariate and LASSO regression. This model includes variables such as age, sex, tumor size, and AJCC stage. The scores for each variable are listed in Table S2. The total score for these factors predicts the probability of all‐cause mortality at 5, 8, and 10 years for ASCC, with higher total scores indicating a worse prognosis. The analysis revealed that the risk of death was higher in male patients compared to females, in unmarried patients compared to married ones, and that the risk increased with advancing age, tumor size, and AJCC stage. For instance, consider a 70‐year‐old male, married, with a 3 cm ASCC tumor, AJCC stage II, who underwent radiochemotherapy without surgery. According to our nomogram, a total risk score of 160 corresponds to a 5‐year survival rate of 48% (95% CI: 43%–55%), an 8‐year survival rate of 36% (95% CI: 30.7%–43%), and a 10‐year survival rate of 29.7% (95% CI: 24.1%–37%).

### 3.4 Validation of Nomogram

**FIGURE 4:**
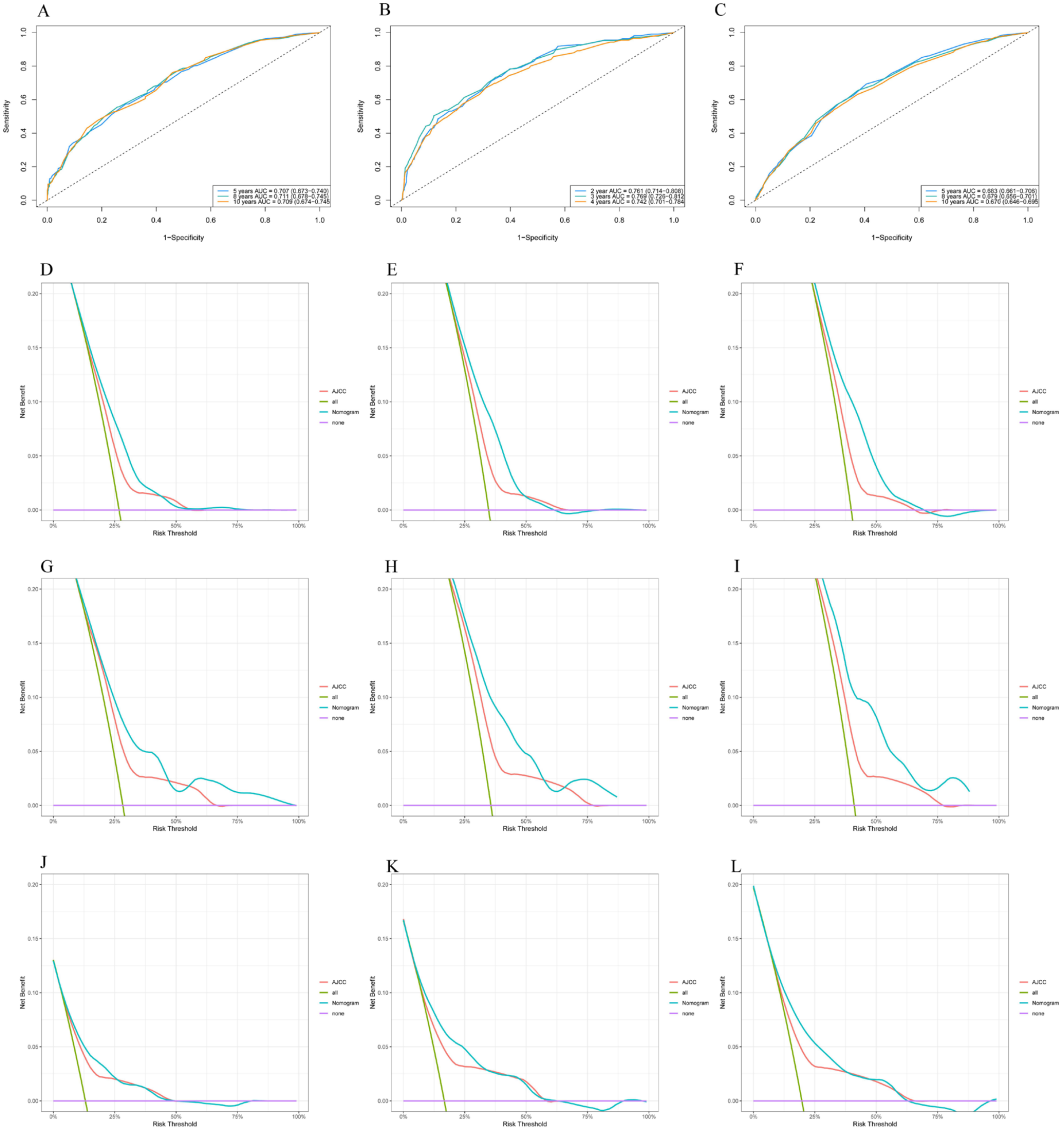
The time‐dependent ROC curves and the DCA curves were plotted based on 5 (2)‐, 8 (3)‐, and 10 (4)‐year OS benefits in the training cohort (C–F), internal validation cohort (A, G–I), and external validation cohort (B, J–L).

**FIGURE 5:**
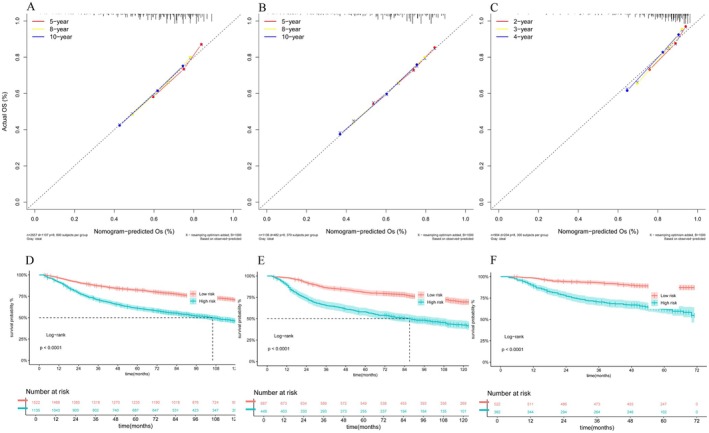
Calibration curves of 5 (2)‐, 8 (3)‐, and 10 (4)‐year OS and Kaplan–Meier curves for OS of ASCC in the low‐risk and high‐risk groups in the training group (A, D), internal validation group (B, E) and external validation group (C, F).

The validation of the constructed nomogram was conducted, and the AUC (Figure 4A–C) for the training cohort was 0.683 (95% CI: 0.661–0.706), 0.679 (95% CI: 0.656–0.701), and 0.670 (95% CI: 0.646–0.695). For the internal validation group, the AUC values were 0.707 (95% CI: 0.673–0.740), 0.711 (95% CI: 0.678–0.745), and 0.709 (95% CI: 0.674–0.745). For the external validation group, the AUC values were 0.761 (95% CI: 0.714–0.808), 0.769 (95% CI: 0.726–0.812), and 0.742 (95% CI: 0.701–0.784). Additionally, the results of the DCA indicated that the prediction model had better clinical applicability (Figure 4D–L). The calibration curve plots for the prognosis prediction model showed excellent agreement between the predicted and actual risks (Figure 5A–C).

### 3.5 Survival Analysis

The cutoff value (87.08) of the risk score in the training group was determined by calculating the individual patient scores on the nomogram, ultimately obtaining a total score for each patient. Based on this, patients were categorized into the low‐risk and high‐risk groups. The subsequent internal and external validation groups were used to assess further the efficacy of this cutoff value in distinguishing between different risk levels. In the training group, there were 1522 low‐risk cases (57.28%) and 1135 high‐risk cases (42.72%). In the internal validation group, there were 687 low‐risk cases (60.48%) and 449 high‐risk cases (39.52%). In the external validation group, there were 522 low‐risk cases (57.74%) and 382 high‐risk cases (42.26%). Kaplan–Meier survival analysis for each risk group showed that the OS of the low‐risk group was significantly better than that of the high‐risk group (*p* < 0.0001), further validating the nomogram‐based model's ability to predict risk scores for ASCC treated with chemoradiotherapy, which has important clinical implications (Figure 5D–F).

### 3.6 Subgroup Analysis

**FIGURE 6:**
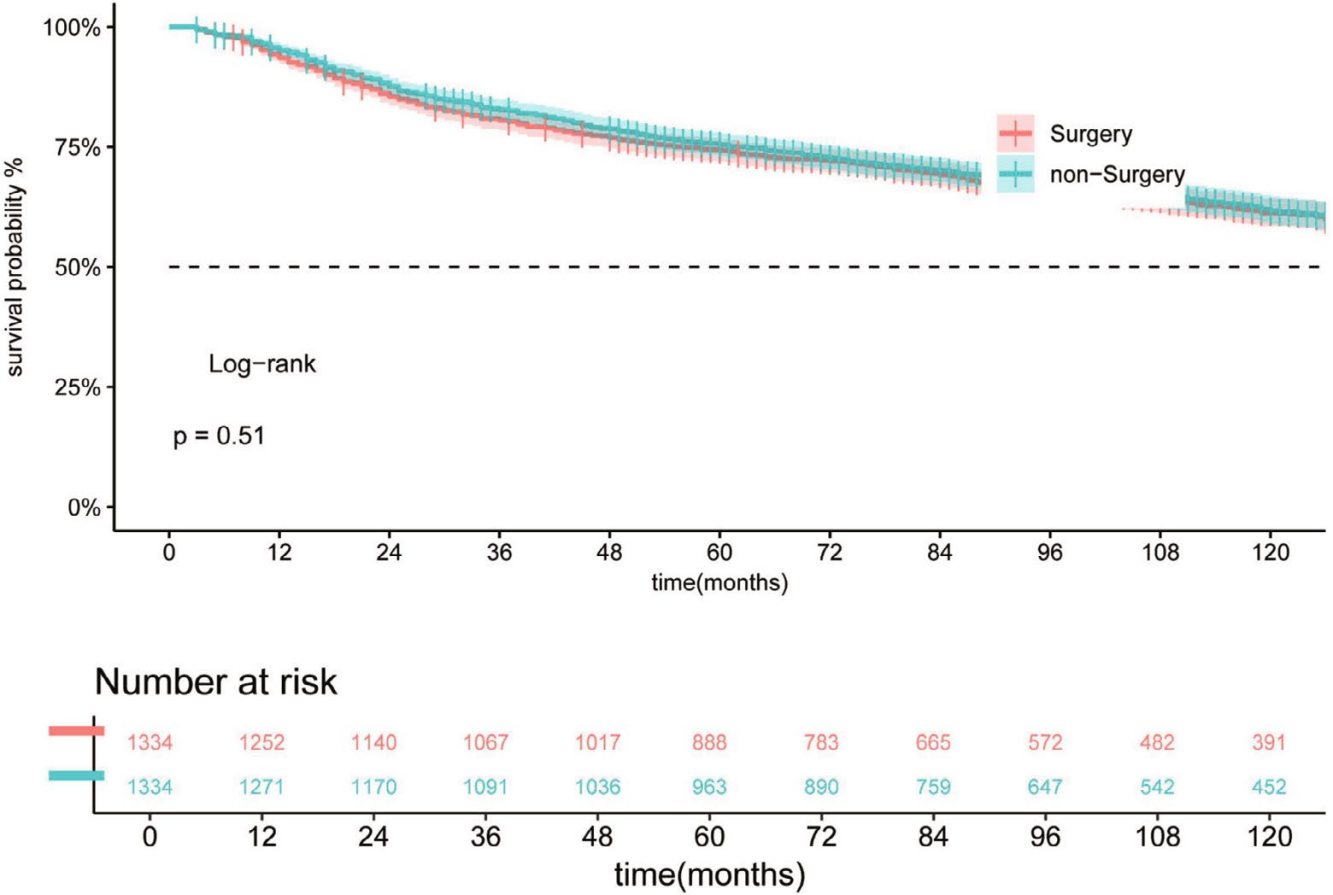
K–M survival curve was plotted according to surgery status after PSM.

**FIGURE 7:**
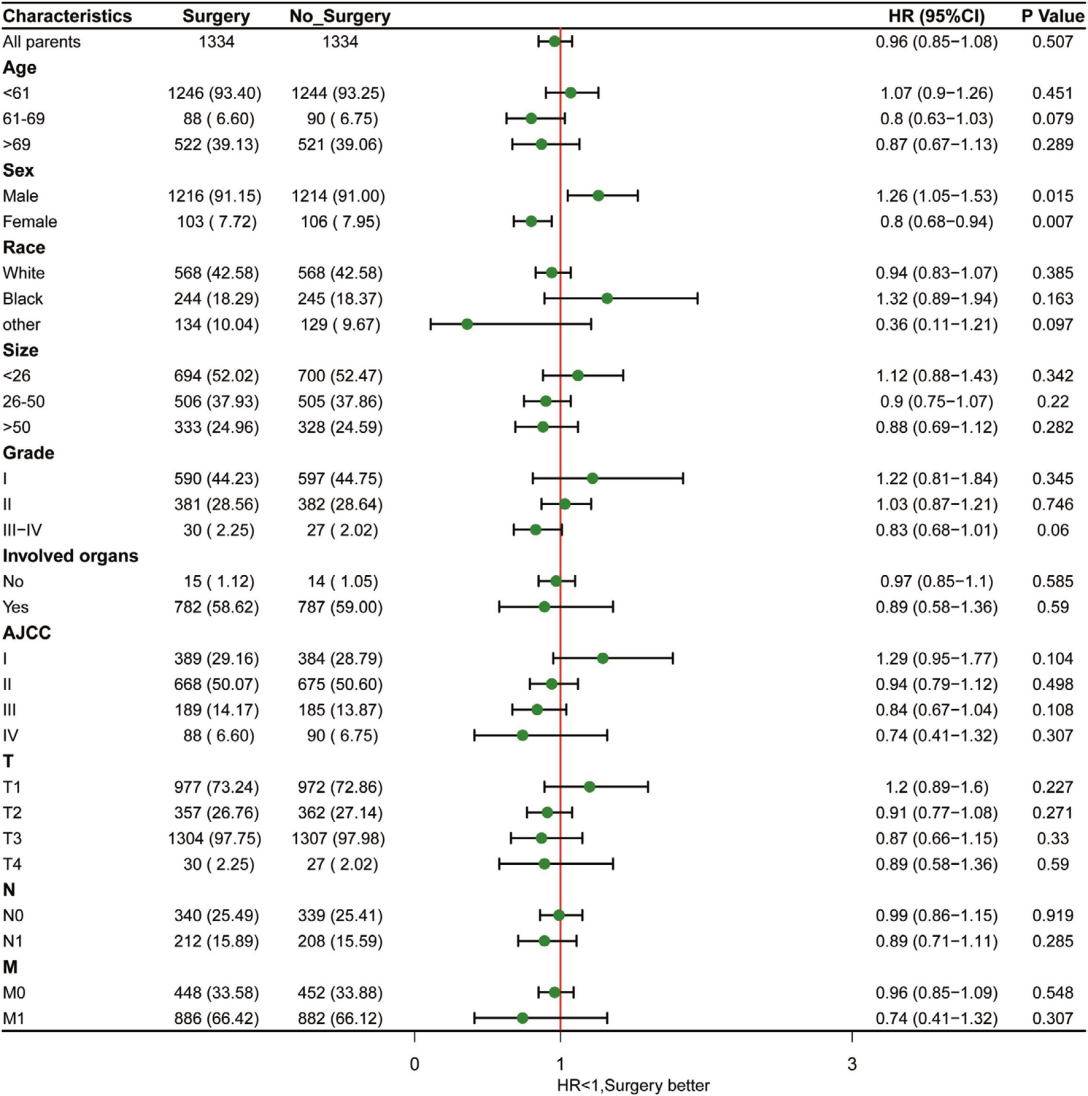
Forest plot for overall survival according to surgery status after PSM.

A K–M survival curve was also plotted based on surgery status, dividing the patients into the surgery and non‐surgery groups in Table 2. PSM was performed to minimize the effect of confounders on the analysis results. A total of 1229 ASCC cases were included in both the surgery and non‐surgery groups. However, no significant improvement in prognosis was observed in the surgery group (Figure 6). To further investigate the potential role of surgery in ASCC treated with chemoradiotherapy, we conducted a subgroup analysis on all patients after PSM (Figure 7). Patients were stratified by age, sex, marital status, race, grade, tumor size, AJCC stage, *T*‐stage, *N*‐stage, and *M*‐stage. We found that surgery significantly improved prognosis in male patients. Interestingly, non‐surgery appeared to be a better management option for female patients. Therefore, gender may serve as an essential criterion for treatment decisions. Additionally, our study indicated that patients with AJCC stage I and well‐differentiated tumors (Grade I) are more likely to benefit from surgery.

**TABLE 2:** Baseline characteristics of ASCC after PSM according to surgery status.

Variables	Levels	Surgery (*n* = 1334)	No‐surgery (*n* = 1334)	*p*
Age (years)	< 61	787 (59.0%)	782 (58.6%)	0.973
	61–69	339 (25.4%)	340 (25.5%)	
	> 69	208 (15.6%)	212 (15.9%)	
Sex	Male	452 (33.9%)	448 (33.6%)	0.902
	Female	882 (66.1%)	886 (66.4%)	
Race	White	1214 (91.0%)	1216 (91.2%)	0.961
	Black	106 (7.9%)	103 (7.7%)	
	Others	14 (1.0%)	15 (1.1%)	
Size (mm)	< 26	521 (39.1%)	522 (39.1%)	0.998
	26–50	568 (42.6%)	568 (42.6%)	
	> 50	245 (18.4%)	244 (18.3%)	
Grade	I	129 (9.7%)	134 (10.0%)	0.941
	II	700 (52.5%)	694 (52.0%)	
	III–IV	505 (37.9%)	506 (37.9%)	
Involved_organs	No	1244 (93.3%)	1246 (93.4%)	0.938
	Yes	90 (6.7%)	88 (6.6%)	
AJCC	I	328 (24.6%)	333 (25.0%)	0.971
	II	597 (44.8%)	590 (44.2%)	
	III	382 (28.6%)	381 (28.6%)	
	IV	27 (2.0%)	30 (2.2%)	
*T*	*T*1	384 (28.8%)	389 (29.2%)	0.987
	*T*2	675 (50.6%)	668 (50.1%)	
	*T*3	185 (13.9%)	189 (14.2%)	
	*T*4	90 (6.7%)	88 (6.6%)	
*N*	*N*0	972 (72.9%)	977 (73.2%)	0.861
	*N*1	362 (27.1%)	357 (26.8%)	
*M*	*M*0	1307 (98.0%)	1304 (97.8%)	0.789
	*M*1	27 (2.0%)	30 (2.2%)	

## 4 Discussion

Nowadays, CRT is the standard treatment for ASCC. Nigro et al. were the first to report that preoperative radiotherapy could significantly improve prognosis, even leading to complete tumor regression, without the need for surgery [18]. Subsequently, several clinical trials have demonstrated CRT efficacy and safety [19, 20]. In our study, age, gender, tumor size, and AJCC stage were identified as independent prognostic factors for ASCC patients receiving chemoradiotherapy through LASSO and multivariate Cox regression analyses. We constructed a nomogram to predict the 5‐year, 8‐year, and 10‐year OS rates. Time‐dependent ROC curve analysis and calibration curves demonstrated favorable performance in both the training and validation sets, confirming the nomogram's ability to accurately predict OS in ASCC patients. DCA further confirmed the superior clinical utility of the nomogram compared to the conventional AJCC staging system. Moreover, the risk‐stratification model effectively categorized patients into the high‐ and low‐risk groups. Notably, our nomogram surpasses the traditional AJCC staging system by incorporating additional factors. We believe this nomogram holds significant potential to accurately predict individual prognoses and guide personalized treatment strategies, ultimately offering meaningful survival benefits to both clinicians and patients.

Previous studies have shown that age, gender, AJCC stage, and radiotherapy were identified as factors for developing a nomogram to better predict patient survival probability [13]. Additionally, Casadei‐Gardini et al. constructed a logistic nomogram that combined laboratory indicators and clinicopathologic features to predict the prognosis of patients receiving concurrent chemoradiation [15]. However, this study included only few patients and incorporated just one clinicopathologic factor—lymph node status—into the nomogram [15], which we believe may explain the small sample size. To the best of our knowledge, this is the first large‐scale retrospective study to utilize a nomogram for survival prediction in ASCC patients undergoing chemoradiotherapy based on SEER data.

In our study, we found that age plays a significant prognostic role in ASCC patients treated with chemoradiotherapy, with prognosis worsening as age increases. This finding is consistent with other studies, including those on different types of tumors [13, 14, 21, 22]. Yang et al. reported that compared to cancer patients aged ≤ 50 years, those aged 50–65 years had a worse prognosis, with the prognosis further deteriorating in patients aged > 65 years [13]. Shao et al. analyzed treatment options for T1/2N0M0 ASCC, noting that more than 80% of their patients were under 70 years old. They concluded that older patients had a worse prognosis [14]. This could be related to the presence of comorbidities in the elderly, decreased sensitivity and tolerance to CRT, and a higher incidence of adverse reactions. The age distribution in their study was similar to ours. We used X‐tile software to group patients by age based on overall survival. However, Leon et al. found that old age was not a significant risk factor for recurrence‐free survival [23]. Despite residual confounding from competing mortality risks (e.g., cardiovascular disease or age‐related comorbidities), our analyses robustly validated the independent prognostic significance of age in ASCC. The heightened prevalence of multiple primary malignancies among elderly ASCC patients (23.3% in our cohort harbored additional primary cancers) introduces complex competing mortality dynamics [24]. Nevertheless, this complexity does not compromise the utility of age for risk stratification. Consequently, integrating age into clinical prognostication facilitates the identification of high‐risk cohorts, enabling tailored therapeutic strategies such as treatment dose attenuation, intensified supportive care, or comprehensive geriatric assessment.

ASCC is more common in women, but males have worse survival outcomes than females [1, 13, 14, 25, 26]. In Japan, female patients comprise 74% of the cases, while in western developed countries, this proportion is approximately 64%. A previous study evaluating international trends in ASCC reported a worldwide rate of about 54% [27, 28]. In our study, female patients represented nearly 70% of the total sample. Our findings showed that females had a better prognosis than males. This may be attributed to the fact that females tend to be more proactive in managing their health, participate in more cancer screenings, and are more likely to be diagnosed at earlier stages of the disease [29, 30].

Tumor size determines the *T*‐stage in ASCC, rather than the depth of tumor infiltration [6, 7]. Claren et al. indicated through multivariate analysis that tumor size is an independent prognostic factor for poorer specific survival [22]. A similar conclusion was reached by Takahashi et al. [26]. Some studies have suggested that tumor size is the most important prognostic factor for disease‐free survival [31, 32]. In other words, the larger the tumor, the more severe the damage to the patient. The prognostic significance of tumor staging is well‐established [13]. Additionally, the utilization of varying AJCC staging system editions across different years within the SEER database introduces potential inconsistencies in staging assignments, thereby impacting the reliability of prognostic predictions. As an iteratively updated framework, the AJCC staging system undergoes periodic revisions to refine staging definitions and incorporate emerging evidence [33, 34, 35]. Notably, studies highlight limitations in the prognostic stratification hierarchy of the AJCC 8th edition for anal cancer, such as the indistinct survival differences within stage III groupings—an issue subsequently addressed in the optimized 9th edition [36]. Specifically, the AJCC 9th edition employs data‐driven methodologies, including survival analysis and validation within large‐scale databases, to refine staging classifications, thereby enhancing prognostic accuracy PEVuZE5vdGU [33, 36]. Consequently, while our nomogram integrates AJCC stage as a key predictor, heterogeneity arising from edition differences, particularly within multi‐edition SEER datasets, may introduce bias. Future studies should prioritize standardization to a single edition or adoption of the most current AJCC criteria to maximize staging comparability.

In our observational cohort study, overall survival was designated as the primary endpoint, aligning with its established role as the most clinically relevant and unbiased metric for assessing long‐term oncologic outcomes in real‐world settings [37, 38]. Large‐scale SEER‐based OS analyses have critically informed recent refinements to the AJCC staging system, as demonstrated by the optimized prognostic stratification in the 9th edition TNM classification for anal carcinoma [37]. This underscores the clinical validity of population‐level OS data. Furthermore, NCCN guidelines emphasize long‐term surveillance for anal cancer patients to monitor disease recurrence and survival endpoints. OS inherently integrates the composite impact of treatment efficacy, tolerance to therapy, treatment‐related complications, and non‐cancer mortality, thereby providing an unambiguous measure of net clinical benefit [6].

Regarding its treatment strategy, chemoradiotherapy is the primary option. With advancements in radiotherapy techniques, one study compared the prognostic impact of 3DCRT and IMRT and found that IMRT was associated with a significant reduction in overall treatment time and improved survival compared to 3DCRT [39]. Receiving treatment at a high‐volume radiation oncology center appears to be linked to improved overall survival in patients with anal tumors. These results may reflect the relationship between physician experience and high‐quality radiotherapy, which is particularly important for rare tumors such as ASCC [40]. However, some studies suggest that treatment options may vary based on staging or risk stratification [14]. Shao et al. argued that low‐risk patients benefited from local resection, moderate‐risk patients benefited from radiotherapy, and high‐risk patients benefited from radiotherapy or chemoradiotherapy [14].

Our nomogram did not include surgery status, as LASSO and Cox multivariate regression analysis determined that surgery was not an independent risk factor for ASCC, which aligns with previous studies [13, 23]. By stratifying patients into the surgery and non‐surgery groups based on surgery status and employing PSM to control for confounding variables, we found that male patients were more likely to benefit from combined chemoradiotherapy and surgery. In contrast, female patients who underwent surgery showed a poorer prognosis. Based on the results of the nomogram and subgroup analyses, it appears that, for male patients, radiotherapy combined with surgery may lead to a better prognosis, although the mechanisms underlying the gender differences require further investigation.

We developed a nomogram that can be used to assess the individualized prognosis of ASCC patients undergoing chemoradiotherapy. However, our study has several notable limitations. First, as a retrospective study based on the SEER database, despite using PSM to minimize intergroup confounders, biases remain inevitable. Second, the SEER database lacks crucial information such as tumor biomarkers, radiotherapy techniques, radiation doses, chemotherapy regimens, and HPV infection status. This absence is particularly significant for ASCC patients, as the optimal radiation dose remains a subject of ongoing debate. Besides, a notable limitation arises from the SEER database's lack of granular detail regarding surgical interventions. While all patients in our institutional cohort received definitive CRT as primary treatment, the SEER registry does not capture the intent of surgery (e.g., salvage procedures following incomplete response or locoregional recurrence after initial CRT) and the adequacy of resection (e.g., non‐radical surgeries requiring adjuvant CRT for positive margins). Finally, the patients included in our study were exclusively from the SEER database. Therefore, future efforts should incorporate data from international databases and conduct prospective clinical trials to validate our nomogram further. Despite these limitations, our nomogram integrates extensive data, offering a valuable tool for predicting OS in ASCC patients undergoing chemoradiotherapy. It also provides invaluable support for developing personalized treatment strategies and making more precise clinical decisions for ASCC patients.

## 5 Conclusion

In conclusion, we developed a nomogram to assess overall survival in ASCC patients treated with chemoradiotherapy, which demonstrated excellent predictive accuracy through validation. We also identified patient populations that may benefit from surgery combined with chemoradiotherapy. This nomogram can assist clinicians in personalizing the management of ASCC and optimizing patient prognosis.

## Author Contributions

**Quan Wang:** methodology, writing – original draft, software. **Guangmin Wan:** methodology, writing – original draft, software. **Lu Yang:** writing – review and editing. **Gang Xu:** conceptualization, project administration, resources, supervision.

## Ethics Statement

The authors have nothing to report.

## Consent

The authors have nothing to report.

## Conflicts of Interest

The authors declare no conflicts of interest.

## Supporting information


Data S1.

## Data Availability

All data can be freely accessed from the SEER program (https://seer.cancer.gov/).
