# Supplementary material for: Prognosis Prediction and Surgical Benefit Subgroup Analysis in Anal Squamous Cell Carcinoma Patients Undergoing Concurrent Chemoradiotherapy
Source: Cancer Med. 2025 Aug 11;14(15):e71091. doi: 10.1002/cam4.71091 (PMC12336998; doi:10.1002/cam4.71091)
Supplement: Supplementary file 1 — Data S1. [file CAM4-14-e71091-s001.docx]

**Prognosis Prediction and Surgical Benefit Subgroup Analysis in Anal Squamous Cell Carcinoma Patients Undergoing Concurrent Chemoradiotherapy**

Quan Wang^[[1]](#footnote-1)^, Guangmin Wan^*^, Lu Yang, Gang Xu^[[2]](#footnote-2)^

Department of Radiation Oncology, The Affiliated Cancer Hospital of Zhengzhou University & Henan Cancer Hospital, Zhengzhou, 450008, China

Table 1 Characteristics of the training group and internal validation group in 2004-2015.

| Variables | training set (n = 2657) | validation set (n = 1136) | P value |
| --- | --- | --- | --- |
| Age (years) |  |  | 0.789 |
| <61 | 1583 (59.58) | 681 (59.95) |  |
| 61-69 | 680 (25.59) | 280 (24.65) |  |
| >69 | 394 (14.83) | 175 (15.4) |  |
| Sex |  |  | 0.667 |
| Male | 865 (32.56) | 361 (31.78) |  |
| Female | 1792 (67.44) | 775 (68.22) |  |
| Race |  |  | 0.911 |
| White | 2357 (88.71) | 1011 (89) |  |
| Black | 233 (8.77) | 99 (8.71) |  |
| Others | 67 (2.52) | 26 (2.29) |  |
| Size (mm) |  |  | 0.384 |
| <26 | 825 (31.05) | 330 (29.05) |  |
| 26-50 | 1211 (45.58) | 543 (47.8) |  |
| >50 | 621 (23.37) | 263 (23.15) |  |
| Grade |  |  | 0.689 |
| I | 274 (10.31) | 127 (11.18) |  |
| II | 1295 (48.74) | 542 (47.71) |  |
| III~IV | 1088 (40.95) | 467 (41.11) |  |
| Involved_organs |  |  | 0.43 |
| No | 2425 (91.27) | 1027 (90.4) |  |
| Yes | 232 (8.73) | 109 (9.6) |  |
| AJCC |  |  | 0.693 |
| I | 494 (18.59) | 203 (17.87) |  |
| II | 1127 (42.42) | 477 (41.99) |  |
| III | 922 (34.7) | 398 (35.04) |  |
| IV | 114 (4.29) | 58 (5.11) |  |
| T |  |  | 0.529 |
| T1 | 597 (22.47) | 237 (20.86) |  |
| T2 | 1342 (50.51) | 591 (52.02) |  |
| T3 | 486 (18.29) | 199 (17.52) |  |
| T4 | 232 (8.73) | 109 (9.6) |  |
| N |  |  | 0.546 |
| N0 | 1739 (65.45) | 742 (65.32) |  |
| N1 | 326 (12.27) | 125 (11) |  |
| M |  |  | 0.308 |
| M0 | 2543 (95.71) | 1078 (94.89) |  |
| M1 | 114 (4.29) | 58 (5.11) |  |
| Surgery |  |  | 0.442 |
| Yes | 939 (35.34) | 386 (33.98) |  |
| No | 1718 (64.66) | 750 (66.02) |  |

Table 2 Each prognostic factor’s score.

| Variable | Levels | Points | Variable | Levels | Points |
| --- | --- | --- | --- | --- | --- |
| Age (years) | <61 | 0 | Sex | Male | 40 |
|  | 61-69 | 21 |  | Female | 0 |
|  | >69 | 66 | AJCC | I | 0 |
| Size (mm) | <26 | 0 |  | II | 23 |
|  | 26-50 | 31 |  | III | 34 |
|  | >50 | 48 |  | IV | 100 |

1. Quan Wang and Guangmin Wan contributed equally to this work. [↑](#footnote-ref-1)
2. Corresponding author: zlyyxugang4124@zzu.edu.cn [↑](#footnote-ref-2)
